# *Enterocytozoon hepatopenaei* (EHP) Infection Alters the Metabolic Processes and Induces Oxidative Stress in *Penaeus vannamei*

**DOI:** 10.3390/ani13233661

**Published:** 2023-11-27

**Authors:** Zheng Cao, Caiyi Chen, Cuixia Wang, Ting Li, Linrui Chang, Lingjun Si, Dongchun Yan

**Affiliations:** Laboratory of Disease Research of Aquatic Animal, School of Agriculture, Ludong University, Yantai 264025, China; caozheng0816@foxmail.com (Z.C.); 13184008178@163.com (C.C.); 17860975326@163.com (C.W.); ttl199071@163.com (T.L.); linruich189@163.com (L.C.)

**Keywords:** *Penaeus vannamei*, microsporidia, glucose metabolism, antioxidant defense, growth performance

## Abstract

**Simple Summary:**

*Enterocytozoon hepatopenaei* (EHP) is highly contagious and has been detected in almost all major shrimp farms in our country. The main symptom of EHP infection is the retarded growth of shrimp and it causes huge economic losses to the aquaculture industry. However, the mechanism of growth retardation remains unclear, so we explored the physiological response mechanisms under EHP experimental infection, including the metabolic processes and oxidative stress. The results showed that EHP infection changed the substance metabolism and growth process. In addition, EHP induced oxidative stress and led to the accumulation of lipid peroxidation products, which may also be an important cause of hepatopancreas tissue damage. It has been observed that severe EHP infection may lead to white feces. This study provides new insights into the subsequent physiological study of host infection with EHP and contributes to the disease prevention and treatment of EHP.

**Abstract:**

*Enterocytozoon hepatopenaei* (EHP) is highly contagious and can cause hepatopancreatic microsporidiosis (HPM), which is typically characterized by the slow growth of shrimp. In this study, the differences in histology, metabolism, oxidative stress and growth between healthy and EHP-infected *Penaeus vannamei* were analyzed using an EHP challenge experiment. Histology showed that EHP caused lesions in the hepatic tubules of *P. vannamei*, such as hepatic tubular atrophy and epithelial cell shedding, with mature spores. Meanwhile, white feces may appear when the infection is severe. Furthermore, the content of total protein, glycogen, ATP and glucose in the EHP challenge group was significantly reduced. The qPCR results showed that EHP infection changed the expression of key genes in glucose metabolism, among which hexokinase (*HK*), phosphofructokinase (*PFK*), pyruvatekinase (*PK*), citrate synthase (*CS*) and isocitric dehydrogenase (*IDH*) were significantly down-regulated, while phosphoenolpyruvate carboxykinase (*PEPCK*), fructose bisphosphatase (*FBP*) and glucose-6-phosphatase (*G6P*) were significantly up-regulated. Obviously, the expression of growth-related genes was disordered. Simultaneously, the antioxidant genes manganese superoxide dismutase (*MnSOD*), catalase (*CAT*), glutathione peroxidase (*GPX*), glutathione-S-transferases (*GST*) and nuclear factor E2-related factor2 (*Nrf2*) were up-regulated to varying degrees in the EHP challenge group, and EHP infection induced significant increases in the oxidative damage products lipid peroxide (LPO) and malondialdehyde (MDA). Ultimately, the shrimp weight of the challenge group was 6.85 ± 0.86 g, which was significantly lower than that of the control group (8.95 ± 0.75 g). Taken together, we speculate that EHP changes the substance metabolism and growth process by causing oxidative damage to the hepatopancreas, which may lead to the growth retardation of *P. vannamei*.

## 1. Introduction

*Penaeus vannamei* (Boone, 1931) is the major shrimp culture species in the world, accounting for the highest proportion of crustacean culture production, and has important economic value [1]. As a major producer of shrimp culture, our country has made an important contribution to the global total shrimp production [2]. However, shrimp are threatened by many diseases in intensive cultivation [3]. Among them, *Enterocytozoon hepatopenaei* (EHP) (Tourtip et al., 2009) is one of the important diseases that reduces production in the process of shrimp culture [4]. It is reported that EHP has been detected in many shrimp culture areas in our country, and the positive rates of EHP in Shandong and Jiangsu have reached 51.2% and 54.40%, respectively, which led to serious economic losses [5,6,7,8]. Internationally, the economic losses caused by EHP in India were about US$ 567.62 million [9], and Shinn et al. estimated that the annual EHP-related losses in Thailand have reached US$ 232 million [10].

Tourtip et al. (2009) described the morphology of EHP in detail for the first time and classified it as a new species of microsporidia [4]. EHP can cause hepatopancreatic microsporidiosis (HPM), resulting in different body sizes and growth retardation of shrimp [11]. At the late stage of EHP infection, shrimp usually exhibit soft shells, reduced food intake, drowsiness and other physiological phenomena. In addition, it has been suggested that there is a relationship between EHP and white feces syndrome, which is characterized by white feces and the detection of EHP spores in the feces of shrimp, and can lead to shrimp death in severe cases [12,13,14]. It is well known that normal material metabolism is a necessary condition to ensure the growth and development of the body. Coincidentally, EHP is mainly parasitic in the hepatopancreas of shrimp. As the infection worsens, the epithelial cells of the hepatic tubular will shed and atrophy [4]. Importantly, as the core metabolic organ of shrimp, the hepatopancreas is responsible for the storage, transportation, absorption and digestion of nutrients [15,16]; its damage affects the metabolic processes of total protein, glucose and glycogen [17,18]. In addition, microspores have lost a large number of metabolism-related genes during their evolution, which means that they are not able to produce enough energy to meet their own needs. Therefore, they are highly dependent on intercepting ATP from the host [19]. Our group and other related omics studies have found that EHP can disrupt the normal glucose metabolism and lipid metabolism of shrimp, change the expression of growth-related genes, and affect the growth and development of shrimp [15,18,20].

As we all know, pathogen invasion can disrupt cellular homeostasis, and then induce abnormal physiological metabolism, mainly by affecting the cellular antioxidant defense system, and even lead to tissue damage. Antioxidant enzymes mainly include superoxide dismutase *(SOD*), catalase (*CAT*), glutathione peroxidase (*GPX*) and glutathione-S-transferase (*GST*), which play an important role in antioxidant stress and repair of oxidative damage. Moreover, lipid peroxidation is an important marker of oxidative stress. Typically, the content of lipid peroxide (LPO) and malondialdehyde (MDA) can reflect the oxidation state of the body [21]. Importantly, when shrimp were infected with *Vibrio parahaemolyticus* (Fujino, 1953) and white spot syndrome virus (WSSV) (Lightner et al. 1996) [22], the expression levels of antioxidant genes in the hepatopancreas were significantly increased [23,24]. It has also been reported that EHP infection can lead to severe oxidative stress in shrimp. However, the pathogen is co-infected with vibrio, so the effect of single EHP infection on the oxidative stress of shrimp remains unclear [25].

In recent years, the research on EHP has mainly focused on infection under natural conditions, and related research has mainly focused on improving the detection methods of EHP [26,27,28]. Hitherto, there has been no research on the effects of EHP infection on the glucose metabolism and oxidative stress of shrimp. Therefore, this study analyzed the changes in key genes of glucose metabolism and antioxidation after *P. vannamei* were infected with EHP via experimental challenge. Simultaneously, related physiological indicators, such as total protein, ATP, glucose and glycogen content, as well as the influence of EHP on shrimp growth performance, were measured. The purpose of this study is to provide evidence for further research on the mechanism of growth retardation of *P. vannamei* under EHP infection by exploring the effects of EHP infection on growth, metabolism and oxidative damage.

## 2. Materials and Methods

### 2.1. Experimental Design and Sample Collection

The *P. vannamei* (3.7 ± 0.3 g) were obtained from a shrimp farm in Weifang City, Shandong Province, China. In order to acclimate the shrimp to the laboratory environment, the shrimp were domesticated in an aquarium (L: 60 cm, W: 50 cm, H: 50 cm) for 10 days and fed twice a day with commercial pellet feed (Tongwei, Chengdu, China). During this period, the shrimp used in the experiments were ensured to be healthy, and randomly selected shrimp were tested for WSSV [29], EHP [5] and infectious hypodermal and hematopoietic necrosis virus (IHHNV) (Lightner, 1983) [30] using PCR. The experimental environment simulated aquaculture ponds with aerated sand-filtered seawater at 25 ± 0.2 °C, salinity 20‰ and pH 7.5 ± 0.2, and half of the seawater was changed twice daily. Healthy shrimp were divided into a challenge group and control group. In the challenge group, 30 shrimp in triplicate were fed with the fresh hepatopancreas of EHP-infected shrimp (EHP copies 10^5^ copies/ng DNA) for 3 d, and the shrimp were fed multiple times a day to keep the shrimp satiated. In the control group, 30 shrimp in triplicate were fed with the fresh hepatopancreas of healthy shrimp for 3 d, and fed multiple times a day to keep the shrimp satiated. After 3 d, both groups of shrimp were fed with commercial feed (Tongwei, China). The shrimp were randomly sampled at the 0th, 5th, 10th and 20th days post-challenge (dpc), and the control group were also collected concurrently. All procedures were in accordance with the guidelines of the respective Animal Research and Ethics Committees of Ludong University and did not involve endangered or protected species.

The sampled tissues were hepatopancreas, muscle and hemolymph. The hepatopancreas and muscle samples were immediately frozen in liquid nitrogen and stored in an ultra-low-temperature refrigerator (−80 °C). The hemolymph was collected with a 1 mL sterile syringe containing anticoagulant (450 mmol/L NaCl, 10 mmol/L KCl, 10 mmol/L EDTA-Na_2_ and 10 mmol/L HEPES, pH 7.45, 780 mOsm/kg) at a ratio of 1:1 (hemolymph:anticoagulant), which was located in the abdominal sinus of the shrimp [31]. After sample collection, direct centrifugation (4 °C) at 800× *g* for 10 min was performed and the supernatant was collected for subsequent experiments.

### 2.2. DNA Extraction and EHP Detection

The total DNA of the shrimp hepatopancreas was extracted using a marine animal tissue genomic DNA extraction kit (DP324, TIANGEN, Beijing, China). EHP-specific primers were used for PCR amplification to identify whether the shrimp were infected with EHP, and the primer sequences were EHP-358F (5′-ATTAGACACCGCTGTAGTTC-3′) and EHP-358R (5′-GTTATTGCCTTCTCCCTC-3′) [5]. The total volume of the PCR reaction was 20 μL, including 10 μL of 2 × Pro Taq Master Mix (AgBio, Changsha, China), 1.0 μL each of the EHP-358 forward and reverse primers, 1.0 μL of DNA template and 7 μL of ddH_2_O. The PCR procedure began at 94 °C for 5 min, followed by 35 cycles of 94 °C for 30 s, 54 °C for 40 s and 72 °C for 40 s and was finally extended for 10 min at 72 °C. To determine whether the shrimp were positive or negative for EHP infection, the PCR products were electrophoresed on 1.0% agarose gels. The EHP load were detected using qPCR using the specific primers EHP185 (F: 5′-GTAGCGGAACGGATAGGG-3′) and EHP185 (R: 5′-CCAGCATTGTCGGCATAG-3′) [32]. The qPCR reaction system consisted of 10 μL of 2 × SYBR qPCR Master Mix (Vazyme, Nanjing, China), 0.4 μL each of the EHP-185 forward and reverse primers (10 μM), 2.0 μL of DNA template and ddH_2_O at a total volume of 20 μL. A total of 40 cycles of the qPCR reaction program were set at 95 °C for 30 s, 95 °C for 10 s and 60 °C for 45 s. A 96-well plate was used for the experiment, and each sample was subjected to three technical and three biological replicates. Gradient dilution was performed according to the constructed plasmid concentration, and a standard curve was established to calculate the EHP load.

### 2.3. Microstructure and Histology

The hepatopancreas at different sampling times was fixed using Davidson fixative [33]. After transparency, embedding, hematoxylin and eosin staining and sealing were carried out, it was placed under an optical microscope (Olympus, IX73SC) for observation.

The white feces were stained using calcofluor white (CFW) dye (Sigma-Aldrich, St. Louis, MO, USA). First, the feces were collected in a 1.5 mL EP tube, homogenized using a grinding rod and centrifuged, and a drop was placed on a glass slide. After removing the excess water, the CFW working solution was added and mixed for dyeing at room temperature, and then the size of spores was observed and calculated using a fluorescence microscope (Olympus, IX73SC) (Image J 2. software) [34].

### 2.4. RNA Extraction and Gene Expression Analysis

Trizol (Invitrogen, Carlsbad, CA, USA) was used to extract the total hepatopancreas. After chloroform extraction, isopropanol precipitation, multiple centrifugation and other steps, the RNA was collected in an enzyme-free EP tube and stored at −80 °C. The RNA quality was evaluated using DeNovix DS-11 (DeNovix, Wilmington, DE, USA) and 1% agarose gel electrophoresis. The cDNA was synthesized using a Fast King RT Kit (with gDNase, TIANGEN, China), according to the commercial instructions and stored at −20 °C for the experiments.

The differences in glucose metabolism, growth-related genes and antioxidant genes between the control group and the challenge group were analyzed using qPCR experiments. They mainly included three key genes, hexokinase (*HK*), phosphofructokinase (*PFK*) and pyruvatekinase (*PK*), of the glycolyic pathway; two key genes, citrate synthase (*CS*) and isocitric dehydrogenase (*IDH*), of the tricarboxylic acid cycle (TCA) and three key enzymes, phosphoenolpyruvate carboxykinase (*PEPCK*), fructose bisphosphatase (*FBP*) and glucose-6-phosphatase (*G6P*), of the gluconeogenic pathway; as well as three growth-related genes, chitinase (*CHI*), ecdysteroid regulated-like protein (*ERP*) and juvenile hormone esterase-like carboxylesterase (*JHEC*). In addition, five antioxidant genes, including *MnSOD*, *CAT*, *GPX*, *GST* and nuclear factor E2-related factor 2 (*Nrf2*), were detected. The Primers were designed using the Primer 5.0 software (Table 1) and synthesized by Sangon (Shanghai, China). The β-actin and ribosome 18s genes were selected as candidate internal reference genes. Since β-actin was evaluated for higher stability with lower variation using the BestKeeper method, it was selected as the housekeeping gene for subsequent analysis. qPCR was performed using an ABI 7500 Fast Real-Time thermal cycler machine (Applied Biosystems, Foster City, CA, USA). The total reaction system was 20 μL, including 10 μL of 2 × qPCR Master Mix (Vazyme, Nanjing, China), 2 μL of cDNA template, 0.4 μL of each primer (10 μM) and 7.2 μL of ddH_2_O. The qPCR procedure was performed as follows: 95 °C for 2 min, followed by 40 cycles of 95 °C for 10 s and 60 °C for 35 s and melting curve analysis at 60–95 °C. All reactions were carried out in triplicate using separate templates. The comparative CT method (2^−ΔΔCT^) was used to analyze the relative expression levels of the genes [35].

### 2.5. Determination of Total Protein, Glycogen, Glucose and ATP Content

The total protein content was determined using a total protein assay kit (Jiancheng, Nanjing, China, No. A045-4). 100 mg of the muscle samples was weighed. The ratio of the sample to PBS was mixed at 1:9, and then the total protein content of the muscle was determined according to the instructions. The measurement process required incubation at 37 °C for 30 min, and the OD value was calculated at 562 nm.

The determination of the hepatopancreas and muscle glycogen content was carried out using a liver/muscle glycogen assay kit (Jiancheng, Nanjing, China, No. A043-1-1). 100 mg of the hepatopancreas and muscle samples was weighed separately. The glycogen detection solution was prepared with a ratio of sample to alkali solution of 1:3, and boiled for 5 min. The OD value was measured and calculated using a wavelength of 620 nm. The above experiments were performed using an Infinite 200 PRO multifunctional enzyme marker (TECAN, Männedorf, Switzerland).

A glucose kit (glucose oxidase method) (Jiancheng, Nanjing, China, No. A154-1-1) was used to analyze the changes in the hemolymph glucose levels. The content of hemolymph glucose was measured directly in accordance with the instructions, and incubated at 37 °C for 10 min. Ultimately, the OD value at 505 nm was evaluated.

The ATP content was measured using an ATP assay kit (Jiancheng, Nanjing, China, No. A095-1-1). The hepatopancreas samples (100 mg) and ddH_2_O were homogenized at a ratio of 1:9, diluted to a concentration of 10%, bathed in water for 10 min and the supernatant was collected for determination after centrifugation. 100 μL of the hemolymph sample was mixed with ddH_2_O at a ratio of 1:4, and the supernatant was also collected using centrifugation for detection. The OD value was measured and calculated using a wavelength of 636 nm.

### 2.6. Detection of Oxidation Products

A lipid peroxidation assay kit (Jiancheng, Nanjing, China, No. A160-1) was used to determine the LPO content. The proportion of hepatopancreas to normal saline was homogenized at 1:9. Incubation was required at 45 °C for 60 min, and the OD value was measured and calculated using a wavelength of 586 nm.

The MDA content was determined using a Malondialdehyde (MDA) Assay Kit (Jiancheng, Nanjing, China, No. A003-1). The tissue homogenate was prepared according to the instructions, incubated at 95 °C for 40 min and the OD value was measured and calculated using a wavelength of 532 nm.

### 2.7. Growth Performance

The growth of the shrimp in the control group and EHP challenge group was recorded, and the relevant growth indicators were calculated. They comprised initial weight, final weight, average daily weight gain (ADG), weight gain, feed conversion ratio (FCR) and survival rate.

The above indicators referred to the method of Zarain-Herzberg et al. [36], where FCR = feed provided/weight gain.

### 2.8. Data Analysis

The data statistics were carried out using the SPSS 17.0 software. One-way analysis of variance and the *t*-test was used to analyze the statistical significance at *p* < 0.05. All numerical data shown in the figures and tables are mean ± SE. Asterisks and double asterisks represented the statistically significant difference and the extremely significant difference between the challenge group and the control group, respectively.

## 3. Results

### 3.1. Detection of EHP Infection

The infection of EHP in the control group and the challenge group was detected using PCR. The shrimp in the challenge group were EHP-positive after the 5th dpc, while the control group remained EHP-negative throughout the experiment. The EHP load was detected using qPCR, and the results showed that the maximum load appeared on the 20th dpc (5.42 × 10^5^ ± 1.51 × 10^5^ EHP copies^−1^ ng HP DNA), followed by the 10th dpc (2.34 × 10^5^ ± 1.25 × 10^5^ EHP copies^−1^ ng HP DNA) and 5th dpc (3.30 × 10^4^ ± 6.19 × 10^3^ EHP copies^−1^ ng HP DNA).

### 3.2. Microscopic Observation and Histology

**Figure 1:**
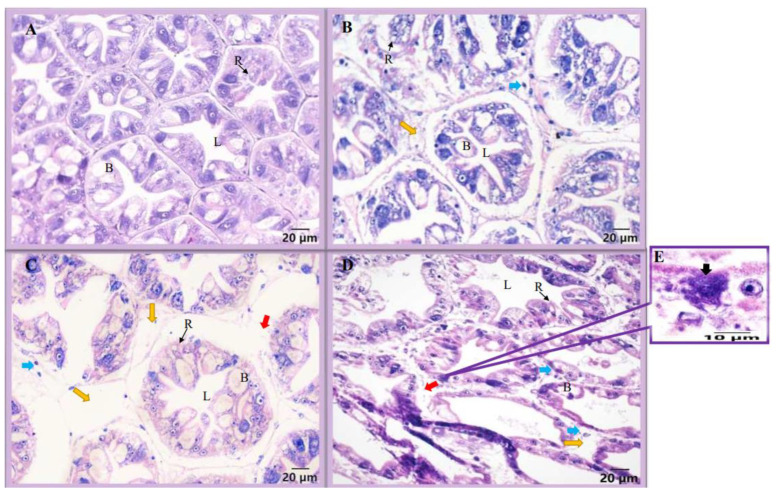
Histology of shrimp hepatopancreas under EHP infection. (**A**) Hepatopancreas of the control shrimp; hepatopancreas of EHP-challenged shrimp at 5th dpc (**B**), 10th dpc (**C**) and 20th dpc (**D**). The blue arrow indicates scattered cells, the yellow arrow indicates vacuoles between hepatic tubules, the red arrow indicates broken basement membrane and the black arrow indicates EHP spores (**E**). B, B cells; L, lumen; R, R cells.

Histological analysis of the hepatopancreas in the control group and the challenge group showed that the hepatic tubules of the healthy hepatopancreas were tightly arranged, with a full structure, a star-shaped lumen and an intact basement membrane (Figure 1A). At the 5th dpc, the hepatopancreas presented with mild atrophy of the hepatic tubules, with exfoliated cells between the tubules and vacuoles between the hepatic tubules (Figure 1B). At the 10th dpc, there were large vacuoles between the hepatic tubules of the hepatopancreas, exfoliated cells between the hepatic tubules and the basement membrane was damaged (Figure 1C). At the 20th dpc, the hepatic tubules showed more serious damage with a looser structure, the basement membrane was severely damaged and the epithelial cells had spore clusters (Figure 1D,E).

**Figure 2:**
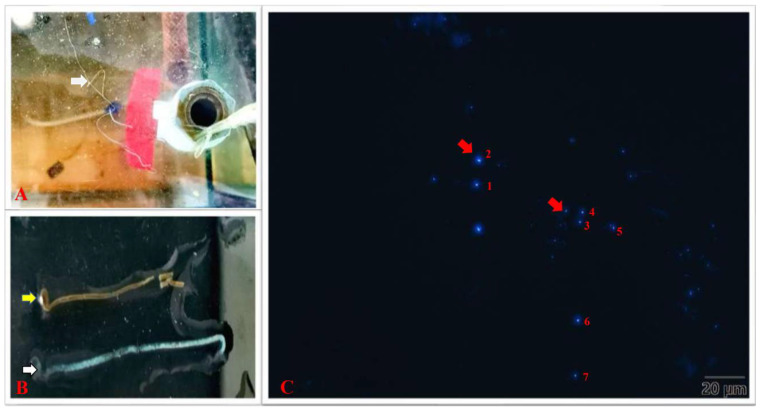
White feces of EHP-infected shrimp and fluorescence microscopic observation of EHP spores in the white feces. (**A**) White feces floating in the aquaculture tank (white arrow); (**B**) the yellow arrow indicates healthy feces, and the white arrow indicates white feces; (**C**) the red arrow indicates EHP spores in white feces.

In addition, when the white feces were stained using CFW working solution, a large number of spores could be observed in the feces (Figure 2C). The size of EHP spores detected in the feces is shown in Table 2.

### 3.3. Total Protein, Glycogen, Glucose and ATP Content Assay

**Figure 3:**
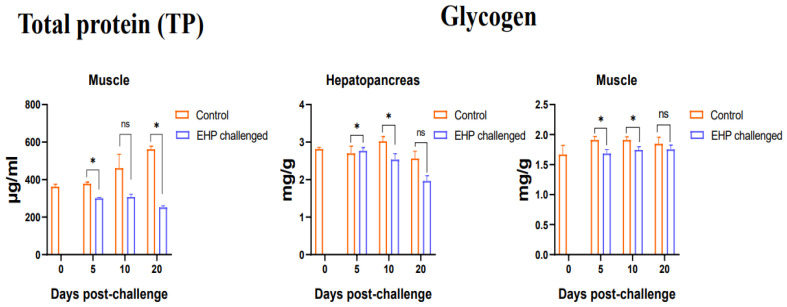
Total protein content in the muscle and glycogen content in the hepatopancreas and muscle of shrimp after EHP infection (mean ± SE, *n* = 6). The superscript symbol * depicts the statistical significance at *p* < 0.05. ns: not significant.

**Figure 4:**
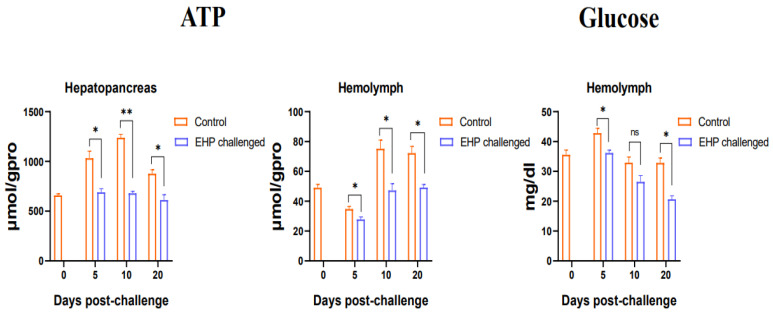
ATP content in the hepatopancreas and hemolymph, and glucose content in the hemolymph of shrimp after EHP infection (mean ± SE, *n* = 6). The superscript symbol * depicts the statistical significance at *p* < 0.05, and ** depicts the statistical significance at *p* < 0.01. ns: not significant.

Compared with the healthy shrimp, the muscle total protein level of the EHP-infected shrimp decreased significantly at the 5th dpc and 20th dpc (*p* < 0.05). At the same time, the results of the determination of the hepatopancreas glycogen and muscle glycogen showed that the hepatopancreas and muscle glycogen of the shrimp in the challenge group decreased significantly at the 5th dpc and 10th dpc (*p* < 0.05), and recovered to the control level at the 20th dpc (Figure 3). Additionally, the results on ATP content showed that the ATP content of the hepatopancreas in the challenge group decreased significantly during the entire experiment (*p* < 0.05), and similarly, the ATP content of the hemolymph also decreased significantly at the 5th dpc, 10th dpc and 20th dpc (*p* < 0.05). Moreover, the hemolymph glucose content of the EHP-infected shrimp decreased significantly at the 5th dpc and 20th dpc (*p* < 0.05) (Figure 4).

### 3.4. Analysis of Glucose Metabolism and Growth-Related Genes

**Figure 5:**
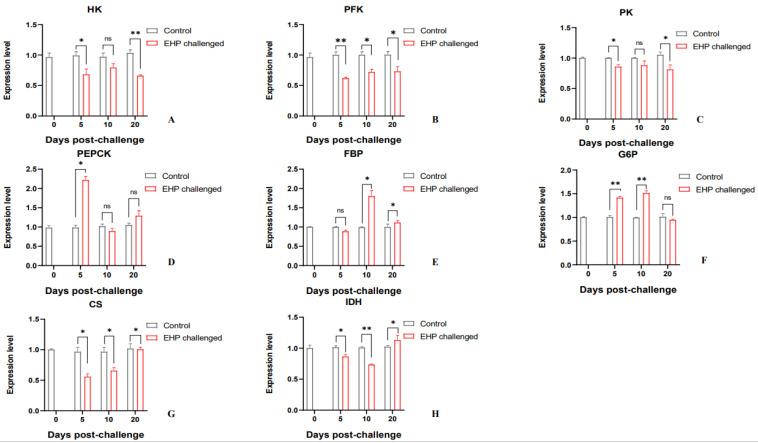
Relative expression levels of glucose metabolism-related genes in the hepatopancreas of *P. vannamei* after EHP infection (mean ± SE, *n* = 6). Glycolysis-related genes: *HK*, *PFK*, *PK* (**A**–**C**); Gluconeogenesis-related genes: *PEPCK*, *FBP*, *G6P* (**D**–**F**); TCA cycle-related genes: *CS*, *IDH* (**G**,**H**). The superscript symbol * depicts the statistical significance at *p* < 0.05, and ** depicts the statistical significance at *p* < 0.01. ns: not significant.

**Figure 6:**
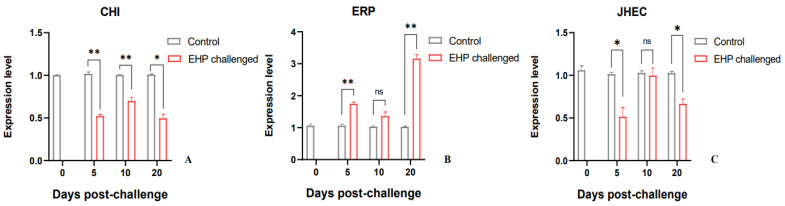
Relative expression levels of growth-related genes *CHI* (**A**), *ERP* (**B**) and *JHEC* (**C**) in the hepatopancreas of *P. vannamei* after EHP infection (mean ± SE, *n* = 6). The superscript symbol * depicts the statistical significance at *p* < 0.05, and ** depicts the statistical significance at *p* < 0.01. ns: not significant.

The expression of key genes in the glucose metabolism pathway was analyzed using qPCR. The qPCR results showed that the key genes of glycolysis in the challenge group were all down-regulated compared to the control group. Among them, *HK* and *PK* were significantly down-regulated at the 5th dpc and 20th dpc (*p* < 0.05), and *PFK* was significantly down-regulated at the 5th dpc, 10th dpc and 20th dpc (*p* < 0.05). The key genes *CS* and *IDH* in the TCA cycle were significantly down-regulated at the 5th dpc, 10th dpc and 20th dpc compared with the control group (*p* < 0.05). However, the key genes in the gluconeogenesis pathway were up-regulated to a certain extent after EHP infection. The expression level of *PEPCK* was increased at the 5th dpc and 20th dpc, of which it was significantly up-regulated at the 5th dpc (*p* < 0.05). The expression of *FBP* increased significantly at the 10th dpc and 20th dpc (*p* < 0.05). *G6P* was significantly up-regulated at the 5th dpc and 10th dpc (*p* < 0.05) (Figure 5). In addition, compared with the control group, the growth-related gene *CHI* was significantly down-regulated during the experiment (*p* < 0.05). *JHEC* was significantly down-regulated at the 5th dpc and 20th dpc (*p* < 0.05). However, *ERP* was significantly up-regulated at the 5th dpc and 20th dpc (*p* < 0.05), but the difference was not significant at the 10th dpc (Figure 6).

### 3.5. Analysis of Antioxidant Genes and Lipid Peroxidation Products

**Figure 7:**
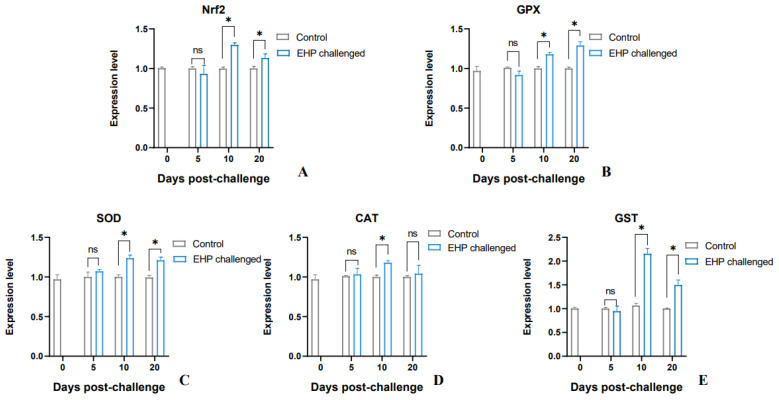
Relative expression levels of antioxidant genes *Nrf2* (**A**), *GPX* (**B**), *SOD* (C), *CAT* (**D**)and *GST* (**E**) in the hepatopancreas of *P. vannamei* after EHP infection (mean ± SE, *n* = 6). The superscript symbol * depicts the statistical significance at *p* < 0.05. ns: not significant.

**Figure 8:**
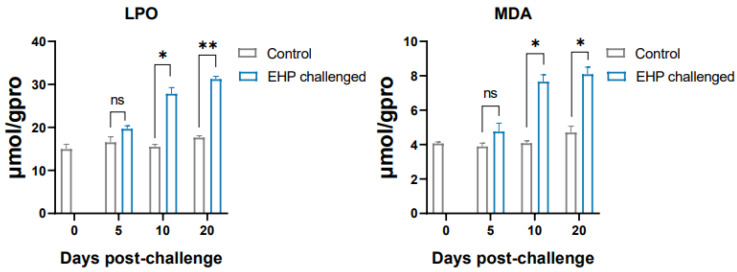
LPO and MDA content in the hepatopancreas of shrimp after EHP infection (mean ± SE, *n* = 6). The superscript symbol * depicts the statistical significance at *p* < 0.05, and ** depicts the statistical significance at *p* < 0.01. ns: not significant.

The results of qPCR showed that the antioxidant enzyme genes *SOD*, *GPX* and *GST* and the antioxidant pathway factor *Nrf2* of the shrimp in the EHP challenge group were significantly up-regulated at the 10th dpc and 20th dpc (*p* < 0.05). However, *CAT* was only significantly up-regulated at the 10th dpc (*p* < 0.05) (Figure 7). Overall, compared with the healthy shrimp, the expression level of antioxidant genes in the EHP-infected shrimp showed an increasing trend. Moreover, the lipid peroxidation products LPO and MDA accumulated significantly at the 10th dpc and 20th dpc (*p* < 0.05) (Figure 8).

### 3.6. Growth Performance

**Figure 9:**
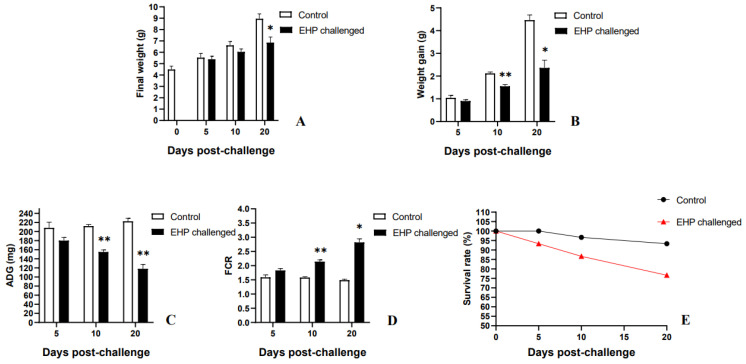
The growth performance of the control group and the EHP challenge group. It mainly includes final weight (**A**), weight gain (**B**), ADG (**C**), FCR (**D**) and survival rate (**E**). The superscript symbol * depicts the statistical significance at *p* < 0.05, and ** depicts the statistical significance at *p* < 0.01.

After 20 days of challenge, the weight of the EHP-infected shrimp was 6.85 ± 0.86 g, which was distinctly lower than that of the control group (8.95 ± 0.75 g). At the same time, the weight gain of the shrimp in the challenge group (2.36 ± 0.34 g) was significantly lower than that in the control group (4.46 ± 0.23 g) (*p* < 0.05). Moreover, the ADG of the shrimp in the challenge group (118.06 ± 17.0 mg) was significantly lower than that in the control group (222.50 ± 11.5 mg) (*p* < 0.05), while the FCR of the challenge group (2.82 ± 0.21) was significantly higher than that in the control group (1.49 ± 0.06) (*p* < 0.05). However, the survival rate of the challenge group (76.67%) was lower than that of the control group (93.34%) (Figure 9). Equally, the growth changes in the shrimp were observed every day during the experiment. At the 7th dpc, shrimp in the challenge group showed a decrease in food intake and activity. At the 16th dpc, it was observed that shrimp in the challenge group showed fecal dragging and slow movement, as well as floating white feces in the breeding tank (Figure 2A,B).

## 4. Discussion

The hepatopancreas of crustaceans integrates the functions of the mammalian liver and pancreas, and is an important organ for nutrient storage, synthesis and digestion. The hepatopancreas, as the main target organ of EHP, even plays an important role in regulating the secretion of growth-related hormones in crustaceans [17,37], which is closely related to the growth retardation caused by EHP. Histology analysis observed that the hepatic tubules of EHP-infected shrimp showed varying degrees of atrophy with the duration of infection, and there were vacuoles between the hepatic tubules, accompanied by the shedding of epithelial cells, which is similar to the results of a previous study [38]. At the 20th dpc, the basement membrane of the hepatic tubules in the challenge group was severely disrupted and EHP spores were observed. At the same time, the EHP load also reached the maximum at the 20th dpc (5.42 × 10^5^ ± 1.51 × 10^5^). This suggests that there is a positive correlation between the degree of hepatopancreas damage and the severity of infection. Compared with a healthy hepatopancreas, these symptoms indicate that the normal absorption and digestion functions of the hepatopancreas are impaired, which may result in the inability of the hepatopancreas to provide nutritional support for the normal growth and development of the shrimp.

As an important nutrient, protein is considered the main source for animal growth and development. This study found that EHP infection caused a decrease in the total protein content of muscle, which may be closely related to the hepatopancreas damage directly caused by EHP parasitism. This further indicates that EHP interfered with the normal muscle development and affected the molting process of the shrimp, resulting in growth retardation [39]. Similarly, infection with WSSV resulted in a decrease in the total protein content in the muscle of shrimp [40], meaning that increasing protein consumption in the body is a self-protection strategy initiated by shrimp against viruses and microsporidia infection. In addition, the significant decrease in ATP content in the hepatopancreas also indicates that the function of the hepatopancreas is damaged and the metabolic process is disordered. Hemolymph is an important component in maintaining a normal metabolism and immune response in shrimp. The content of ATP and glucose in the hemolymph decreased, indicating that EHP infection disrupted the normal metabolic process and increased energy consumption, which is consistent with the findings of previous research [37]. Analogously, it has been reported that after shrimp are infected with WSSV, the ATP content in the hemolymph decreased significantly as the infection time was prolonged [41,42]. Meanwhile, the glucose content in the hemolymph can be regarded an important physiological indicator of shrimp health. As an important energy storage substance, glycogen can rapidly provide energy to the body under stress and starvation. It has been suggested that microsporidia act as a “carbohydrate collector”, absorbing glucose from the host and forcing the hepatopancreas to mobilize stored carbohydrates [43,44]. In the present study, it was found that the glycogen levels in the hepatopancreas and muscle of shrimp decreased significantly after EHP infection. Likewise, a significant reduction in glycogen content in the hepatopancreas has also been reported in the Norway lobster *Nephrops norvegicus* (Linnaeus, 1758) infected with parasitic dinoflagellates [44]. It is possible that EHP starves the shrimp during infection and consumes the glycogen of the host, as if EHP is issuing instructions to the shrimp that require large amounts of energy. Moreover, impaired hepatopancreatic function is associated with severe infection of glycogen storage R cells and secretory B cells [45,46].

Whether carbohydrate metabolism is normal or not can affect the molting and growth process of crustaceans, as well as the ability to cope with hypoxia stress and the invasion of pathogenic microorganisms [47,48,49]. Critically, changes in key enzymes in carbohydrate metabolism are the decisive factor in determining whether a certain metabolic pathway is able to proceed normally. Among them, glycolysis, the TCA cycle and gluconeogenesis are the basic pathways of glucose metabolism in eukaryotes. Overall, the present study showed that EHP infection suppressed the gene expression levels of key regulatory enzymes in glycolysis and the TCA cycle, possibly reducing the metabolic rate, which is consistent with previous studies [15,18,20,50]. However, the gene expression of key regulatory enzymes in the gluconeogenesis pathway was significantly up-regulated. *HK*, *PFK* and *PK* are important key regulatory enzymes in the glycolytic pathway, and changes in their expression affect the entire pathway [51]. The expression of key enzymes in the glycolysis pathway decreased significantly after EHP infection, indicating that the metabolic pathway was inhibited. Among these enzymes, *PFK* is considered to play a restrictive role in the pathway, determining the rate and direction of the reaction [52,53]. EHP significantly inhibited the gene expression of *PFK*, which means that the capacity of anaerobic metabolism decreased, the metabolic rate slowed down and the metabolic process changed. Meanwhile, the key regulatory enzymes in the TCA cycle, *CS* and *IDH*, were significantly down-regulated. As the primary key regulatory enzyme of the TCA cycle, *CS* controls the speed of acetyl-CoA entering the TCA cycle and releasing ATP via oxidative phosphorylation [54]. *IDH*, another key regulatory enzyme in the TCA cycle, catalyzes the oxidative decarboxylation of isocitrate into 2-oxoglutarate and releases ATP via the electron transport chain [55]. The decrease in the expression level of key enzymes in the TCA cycle may be due to the inhibition of the upstream glycolysis reaction by EHP, resulting in fewer products entering the TCA cycle, precisely because the parasitism of EHP disrupts the glucose metabolic process of the host. Glycolysis is an important way for crustaceans to obtain energy. As the core of carbohydrate, protein and lipid metabolism, the TCA cycle is the main source of cellular energy [56]. If the activity of its key enzyme is inhibited, it will directly cause a decrease in ATP production, resulting in a shortage of energy necessary for cell development. This may be an important reason for the slow growth of shrimp. Therefore, host cells may adopt strategies to reduce enzyme activity and decrease the metabolic intensity to maintain the host’s survival for as long as possible in the presence of prolonged infection. In addition, the glycolysis and TCA cycle metabolic processes were impaired by EHP, while the key regulatory enzymes in the gluconeogenesis pathway tended to increase. *FBP* is a rate-limiting enzyme for gluconeogenesis, which controls the rate of gluconeogenesis and catalyzes the conversion of fructose-1,6-diphosphate into fructose-6-phosphate [51]. After EHP infection, *FBP* expression was significantly increased at the 10th dpc and 20th dpc, and the expression of key enzymes *PEPCK* and *G6P* was also up-regulated, indicating that the gluconeogenesis process was enhanced. Since the parasitism of EHP needs to continuously absorb the energy from the host to maintain its own proliferation, the host is in a state of starvation [11]. This situation has also been observed in bees infected with parasites [57]. It is possible that the organism can maintain a normal hemolymph glucose level in shrimp by enhancing gluconeogenesis, but the degree of enhancement is not enough to compensate for the glucose consumption caused by EHP parasitism. Therefore, the hemolymph glucose content was still lower than that of the control group. Meanwhile, the consumption of carbohydrates was greater than the intake, which may be another reason for the decrease in glucose content. Interestingly, when shrimp were infected with WSSV [58] or *Vibrio parahaemolyticus*, the glucose metabolism process in vivo was significantly enhanced [59]. WSSV infection may be similar to the Warburg effect by enhancing glucose metabolism in response to viral replication [40,60]. Perhaps the study of changes in key metabolites can be regarded as a way to distinguish between viral infections and pathogenic microbial infections.

Oxidative stress is induced by excessive accumulation of free radicals and reactive oxygen species (ROS) in the body, which is an important factor leading to apoptosis and disease. It can be caused by stressors in vivo or in vitro. In order to prevent the damage caused by ROS, organisms have evolved an antioxidant defense system, with enzymatic responses to oxidative stress as the main strategy [61]. Therefore, a series of antioxidant enzymes play an important role, such as *SOD*, *CAT*, *GPX* and *GST* [62]. *MnSOD* is mainly distributed in the mitochondria and can catalyze the decomposition of superoxide into oxygen and hydrogen peroxide. Previous results found that the transcription level of *MnSOD* increased significantly after shrimp were injected with the pathogen [63]. Similarly, in this study, significant increases in the expression levels of *MnSOD* and *GPX* were detected at the 10th dpc and 20th dpc, suggesting that EHP activated the antioxidant defense system of the shrimp. In addition, the body produces a large amount of ROS, which can induce the expression of antioxidant genes, and in turn protect host cells to reduce oxidative damage [64]. Usually, the change in *CAT* is consistent with that in *SOD*, which is responsible for the decomposition of hydrogen peroxide into oxygen and water. However, *CAT* was significantly elevated only at the 10th dpc. It may be because both *CAT* and *GPX* perform the function of decomposing peroxides, but *GPX* is more sensitive to peroxides in the body and can play a role even at low concentrations. This was verified using nitrite stress experiments [65,66]. Meanwhile, *GPX* has the function of detoxification metabolism, which is considered to be more important than *CAT*. Importantly, *GPX* in shrimp can be induced by *Vibrio alginolyticus* [67]. The expression of *GST* also increased significantly in this experiment, suggesting that it may participate in the transport of endogenous substances and play an irreplaceable role in detoxification and cell protection [68]. The *Nrf2-Keap1* signaling pathway is the most important antioxidant pathway in the body, and can regulate the activity of antioxidant enzymes [69]. Unfortunately, Keap1 has not been identified in *P. vannamei*. It has been reported that inhibiting the expression of *Nrf2* resulted in a significant decrease in the expression of related antioxidant genes (*SOD*, *GPX*, *CAT*) in shrimp [70]. In this study, *Nrf2* was significantly up-regulated after EHP infection, which was consistent with the expression of antioxidant enzymes, indicating that this gene may regulate the expression of antioxidant genes. *Nrf2* has also been reported to be significantly activated in the response of *P. vannamei* to *Vibrio harveyi* [71]. In addition, LPO is a lipid peroxidation product generated by the reaction of ROS with polyunsaturated fatty acids, which causes oxidative damage to cells. MDA is also the result of lipid peroxidation and is considered to be a marker of oxidative damage [72]. In organisms, polyunsaturated fatty acids are the main components of the membrane structure that make up various organelles. The increase in LPO and MDA content detected in this experiment after EHP infection suggests that the hepatopancreas cells may be damaged and interfere with normal physiological functions. Corresponding to the histology, it was clearly observed that the hepatopancreas was significantly damaged, with vacuoles and membrane damage between the hepatic tubules, which may be tissue damage caused by a large number of peroxide products. These results reveal that the activation of the antioxidant pathway and the up-regulated expression of antioxidant oxidase genes induced by EHP are not sufficient to clear excess lipid peroxidation products, and the tissue damage caused by oxidative stress may be the key reason that affects the metabolic function of the hepatopancreas.

The present study also demonstrated that EHP can lead to the abnormal expression of growth-related genes, among which *JHEC*, *ERP* and *CHI* play important regulatory roles in molting, growth and reproduction in crustaceans. In this experiment, *JHEC* (at the 5th dpc and 20th dpc) was significantly down-regulated, whereas *ERP* (at the 5th dpc and 20th dpc) was significantly up-regulated. *JHEC* is a key enzyme in the degradation of methyl farnesoate (MF) [73]. The reduction of the MF titer is beneficial to the growth and development of shrimp. On the contrary, the down-regulation of *JHEC* reduced the degradation rate of MF, resulting in stunted growth and development. The increase in ecdysterone is conducive to molting, but there is a negative regulatory relationship between *ERP* and ecdysterone. When the level of ecdysterone is high, the expression of *ERP* is inhibited, and the up-regulation of *ERP* in the present study was manifested as a delayed molting process [74,75]. Concurrently, *CHI* can digest chitin, and the enhanced activity of this enzyme before molting can ensure a smooth molting process by decomposing the old shell [76,77]. However, in this study, EHP invasion resulted in a significant decrease in *CHI*, indicating that shrimp molting was blocked. Therefore, the parasitism of EHP destroys the normal substance metabolism in shrimp, resulting in insufficient energy for cell growth and development. Furthermore, EHP invasion breaks the balance between growth and molting-related hormones, which may be an important cause of retarded development of shrimp.

EHP can significantly damage the hepatopancreas of shrimp, disrupt the normal intestinal flora and cause nutrient absorption and transport disorders. Therefore, the final body weight and ADG of shrimp in the EHP challenge group were significantly lower than those in the control group. Similarly, this phenomenon of different individual sizes is more obvious in aquaculture ponds [78]. In addition, the FCR of shrimp in the EHP challenge group was significantly higher than that in the control group (20th dpc) (*p* < 0.05) due to the disruption of nutrient absorption and transport, indicating that the shrimp infected with EHP could not adequately absorb the nutrients in the feed. Fortunately, the administration of 5-aminolaevulinic acid and linolenic acid to EHP-infected shrimp could improve the function of the hepatopancreas to a certain extent [75,79]. In addition, EHP infection did not cause a large number of deaths in shrimp, but increased the risk of infection with other diseases such as acute hepatopancreatic necrosis disease (AHPND) (Lightner et al. 2012) [12], taura syndrome [80] and white feces syndrome [13]. Previous studies have shown that white feces can be present with the aggravation of EHP infection, but white feces are not real feces and may be composed of EHP spores, intestinal mucus and necrotic tubular epithelial cells. Many studies have shown that EHP spores have been detected in white feces [13,81]. Consistent with previous reports, floating white feces were also found in the tanks of EHP-challenged shrimp (at the 16th dpc). A large number of spores were observed after the suspended feces were stained, indicating that the phenomenon of white feces has a certain relationship with EHP infection.

## 5. Conclusions

In this study, the changes in hepatopancreas histology and the effects of EHP on the metabolism, oxidative stress and growth-related genes of healthy shrimp and EHP-infected shrimp were analyzed using EHP challenge experiment. Our studies have shown that the hepatopancreas became damaged with an increasing duration of EHP infection, and the degree of damage was positively correlated with the intensity of infection. At the same time, EHP infection affected the normal metabolism of substances and growth, which was characterized by the inhibition of glycolysis and the TCA cycle, activation of the gluconeogenesis pathway, and interference with the expression of growth-related genes. Furthermore, EHP induced oxidative stress in shrimp and resulted in the accumulation of a large amount of the lipid peroxidation products LPO and MDA, which was also an important cause of oxidative damage to the hepatopancreas. Additionally, severe EHP infection may result in white feces. In summary, this study concludes that EHP infection can disrupt the metabolic process of shrimp and cause oxidative stress, both of which will lead to the slow growth of shrimp and even death in severe cases.

## Figures and Tables

**Table 1:** Primers used for qPCR in the experiment.

Gene	Primers (5′-3′)	GenBank Accession No.
Growth and metabolic genes		
*ERP*	F: CCTACAGCGTCAACATCCA R: GCATCCGTCGGTGTCTATT	JQ009182.1
*JHEC1*	F: GGCGGAGCAGAGGACTAT R: CGAGGTCACGGATGTTGTC	APO14259.1
*CHI*	F: GAAGGCGTCGTATATTGTGTCAC R: AGCACGGTCCTGATGGTAGT	ACR23315
*G6P*	F: CTTATGAATCGTGGGTGGTGC R: CAATAGGGTCGGTCTCCTCTGA	PRJNA660490
*FBP*	F: TGTGTCGGAAGAAAACAAAACT R: CTATGGAGACGAGGCAATCAAT	KP057246
*PEPCK*	F: AAGACCAGTGATGGAGGAGTG R: GGGAGTTGGGATGAGCAG	FJ441189
*PK*	F: ATCCTTGATGGTGCTGAC R: TGAAGAGTTGCTTGTGCC	EF102105
*PFK*	F: AGAGGACGGGGAAGTTTTACAG R: GTTCTTGCCTGGGTTCAAATAG	PRJNA660490
*HK*	F: ATCGGCAAGTTAGATACGC R: AGGACACCACGGTAGGAA	EF102106
*CS*	F: AAAGAATACGGTAACACCAAAGT R: ATGGAGTAACCTCGGAACCTGAT	XM027367291
*IDH*	F: GCCTCTGCCTCAAGGGTAT R: CCTCAGTCTGCTCACGGAT	XM027354101.1
Antioxidant genes		
*CAT*	F: TCAGCGTTTGGTGGAGAA R: GCCTGGCTCATCTTTATC	AY518322
*MnSOD*	F: CGTAGAGGGTATTGTCGT R: TTGAAATCATACTTGAGGG	DQ005531
*GPX*	F: AGGGACTTCCACCAGATG R: CAACAACTCCCCTTCGGTA	AY973252
*GST*	F: AAGATAACGCAGAGCAAGG R: TCGTAGGTGACGGTAAAGA	AY573381
*Nrf2*	F: GATGAAGCGAGCCAGAGCG R: GCCGTCGGATGTCTCGGATAA	XM_027367068.1
*β-actin*	F: TGGACTTCGAGCAGGAGATG R: GGAATGAGGGCTGGAACAGG	AF300705
*18s*	F: TATACGCTAGTGGAGCTGGAA R: GGGGAGGTAGTGACGAAAAAT	EU920969

**Table 2:** The size of EHP spores in the experiment.

**Number**	1	2	3	4	5	6	7
**Size (μm)**	1.102	1.193	0.928	0.717	1.009	1.055	0.820

## Data Availability

The datasets used in this study are available from the corresponding authors upon reasonable request.
